# Genetic Determinants of Circulating Sphingolipid Concentrations in European Populations

**DOI:** 10.1371/journal.pgen.1000672

**Published:** 2009-10-02

**Authors:** Andrew A. Hicks, Peter P. Pramstaller, Åsa Johansson, Veronique Vitart, Igor Rudan, Peter Ugocsai, Yurii Aulchenko, Christopher S. Franklin, Gerhard Liebisch, Jeanette Erdmann, Inger Jonasson, Irina V. Zorkoltseva, Cristian Pattaro, Caroline Hayward, Aaron Isaacs, Christian Hengstenberg, Susan Campbell, Carsten Gnewuch, A. CecileJ.W. Janssens, Anatoly V. Kirichenko, Inke R. König, Fabio Marroni, Ozren Polasek, Ayse Demirkan, Ivana Kolcic, Christine Schwienbacher, Wilmar Igl, Zrinka Biloglav, Jacqueline C. M. Witteman, Irene Pichler, Ghazal Zaboli, Tatiana I. Axenovich, Annette Peters, Stefan Schreiber, H.-Erich Wichmann, Heribert Schunkert, Nick Hastie, Ben A. Oostra, Sarah H. Wild, Thomas Meitinger, Ulf Gyllensten, Cornelia M. van Duijn, James F. Wilson, Alan Wright, Gerd Schmitz, Harry Campbell

**Affiliations:** 1 Institute of Genetic Medicine, European Academy Bozen/Bolzano (EURAC), Bolzano, Italy, Affiliated Institute of the University of Lübeck, Lübeck, Germany; 2 Department of Neurology, General Central Hospital, Bolzano, Italy; 3 Department of Neurology, University of Lübeck, Lübeck, Germany; 4 Department of Genetics and Pathology, Rudbeck Laboratory, Uppsala University, Uppsala, Sweden; 5 MRC Human Genetics Unit, IGMM, Western General Hospital, Edinburgh, United Kingdom; 6 Centre for Population Health Sciences, University of Edinburgh, Edinburgh, United Kingdom; 7 Croatian Centre for Global Health, Faculty of Medicine, University of Split, Split, Croatia; 8 Gen-info Ltd, Zagreb, Croatia; 9 Institute for Clinical Chemistry and Laboratory Medicine, University Hospital Regensburg, Regensburg, Germany; 10 Department of Epidemiology, Erasmus University Medical Center, Rotterdam, The Netherlands; 11 Medizinische Klinik II, University of Lübeck, Lübeck, Germany; 12 Institute of Cytology and Genetics SD RAS, Novosibirsk, Russia; 13 Klinik und Poliklinik für Innere Medizin II, Universität Regensburg, Regensburg, Germany; 14 Institut für Medizinische Biometrie und Statistik, University of Lübeck, Lübeck, Germany; 15 Andrija Stampar School of Public Health, Faculty of Medicine, University of Zagreb, Zagreb, Croatia; 16 Department of Experimental and Diagnostic Medicine, University of Ferrara, Ferrara, Italy; 17 Institute of Epidemiology, Helmholtz Zentrum München – German Research Center for Environmental Health, Neuherberg, Germany; 18 Institut für Klinische Molekularbiologie, Christian-Albrechts Universität, Kiel, Germany; 19 Institute of Medical Information Science, Biometry and Epidemiology, Chair of Epidemiology, LMU Munich, Germany; 20 Department of Clinical Genetics, Erasmus University Medical Center, Rotterdam, The Netherlands; 21 Helmholtz Zentrum München, Neuherberg, Munich, Germany; The University of Queensland, Australia

## Abstract

Sphingolipids have essential roles as structural components of cell membranes and in cell signalling, and disruption of their metabolism causes several diseases, with diverse neurological, psychiatric, and metabolic consequences. Increasingly, variants within a few of the genes that encode enzymes involved in sphingolipid metabolism are being associated with complex disease phenotypes. Direct experimental evidence supports a role of specific sphingolipid species in several common complex chronic disease processes including atherosclerotic plaque formation, myocardial infarction (MI), cardiomyopathy, pancreatic β-cell failure, insulin resistance, and type 2 diabetes mellitus. Therefore, sphingolipids represent novel and important intermediate phenotypes for genetic analysis, yet little is known about the major genetic variants that influence their circulating levels in the general population. We performed a genome-wide association study (GWAS) between 318,237 single-nucleotide polymorphisms (SNPs) and levels of circulating sphingomyelin (SM), dihydrosphingomyelin (Dih-SM), ceramide (Cer), and glucosylceramide (GluCer) single lipid species (33 traits); and 43 matched metabolite ratios measured in 4,400 subjects from five diverse European populations. Associated variants (32) in five genomic regions were identified with genome-wide significant corrected *p*-values ranging down to 9.08×10^−66^. The strongest associations were observed in or near 7 genes functionally involved in ceramide biosynthesis and trafficking: *SPTLC3*, *LASS4*, *SGPP1*, *ATP10D*, and *FADS1–3*. Variants in 3 loci (*ATP10D*, *FADS3*, and *SPTLC3*) associate with MI in a series of three German MI studies. An additional 70 variants across 23 candidate genes involved in sphingolipid-metabolizing pathways also demonstrate association (*p* = 10^−4^ or less). Circulating concentrations of several key components in sphingolipid metabolism are thus under strong genetic control, and variants in these loci can be tested for a role in the development of common cardiovascular, metabolic, neurological, and psychiatric diseases.

## Author Summary

Although several rare monogenic diseases are caused by defects in enzymes involved in sphingolipid biosynthesis and metabolism, little is known about the major variants that control the circulating levels of these important bioactive molecules. As well as being essential components of plasma membranes and endosomes, sphingolipids play critical roles in cell surface protection, protein and lipid transport and sorting, and cellular signalling cascades. Experimental evidence supports a role for sphingolipids in several common complex chronic metabolic, cardiovascular, or neurological disease processes. Therefore, sphingolipids represent novel and important intermediate phenotypes for genetic analysis, and discovering the genetic variants that influence their circulating concentrations is an important step towards understanding how the genetic control of sphingolipids might contribute to common human disease. We have identified 32 variants in 7 genes that have a strong effect on the circulating plasma levels of 33 distinct sphingolipids, and 43 matched metabolite ratios. In a series of 3 German MI studies, we see association with MI for variants in 3 of the genes tested. Further cardiovascular, metabolic, neurological, and psychiatric disease associations can be tested with the variants described here, which may identify additional disease risk and potentially useful therapeutic targets.

## Introduction

Sphingolipids are essential components of plasma membranes and endosomes and are believed to play critical roles in cell surface protection, protein and lipid transport and sorting, and cellular signalling cascades. They are known to have roles in both health and disease [1],[2]. Several rare monogenic diseases associated with sphingolipid biosynthesis and turnover have been identified such as metachromatic leukodystrophy and GM1- and GM2-gangliosidosis, Niemann-Pick, Gaucher, Krabbe, Fabry, Farber, Tay-Sachs and Sandhoff diseases [3]. Defective biosynthesis due to mutations in genes involved in sphingolipid metabolism (e.g.serine palmitoyl transferase (*SPTLC1*) [4]; ceroid-lipofuscinosis, neuronal 8 (*CLN8*) [5]; and ceramide synthase (*LASS1*) [6]) can also lead to disease. Moreover, natural fungal inhibitors of ceramide synthase can result in a broad spectrum of effects including equine leucoencephalomalacia, porcine pulmonary oedema syndrome and liver cancer in rats [7], demonstrating the wide range of processes that include cell proliferation, differentiation and apoptosis underpinned by sphingolipid metabolism. Identifying common genetic variants that influence the balance between individual sphingolipid concentrations represents an important step towards understanding the contribution of sphingolipids to common human disease. To achieve this goal, we conducted a genome-wide association study (GWAS) on plasma levels of 33 major sphingolipid species (24 sphingomyelins and 9 ceramides) in five European populations, both within and across populations. The traits were analysed by individual species (sphingomyelins (SM), dihydrosphingomyelins (Dih SM), ceramides (Cer) and glucosylceramides (GluCer)) or aggregated into groups of species with similar characteristics (e.g. unsaturated ceramides), and expressed as absolute concentrations or as molar percentages within sphingolipid classes (mol%). In addition we examined 43 matched metabolite ratios between the traits as a surrogate for enzyme activity [8] in separate clusters designed to examine sphingolipid metabolism (11 ratios), desaturation (16 ratios) and elongation (16 ratios). All traits displayed substantial heritabilities in that much of the observed variation in sphingolipid levels could be attributed to genetic variation among individuals in each population.

## Results

**Figure 1:**
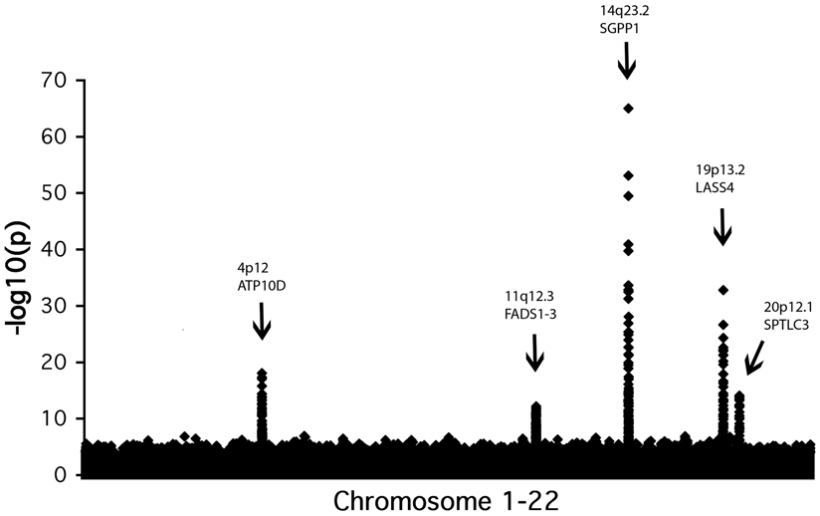
Genome-wide association results for sphingolipids. Manhattan plots show the association signals (−log10 of *p*-value) on the y-axis versus SNPs according to their position in the genome on the x-axis (build 36). The most interesting candidate genes are highlighted.

**Figure 2:**
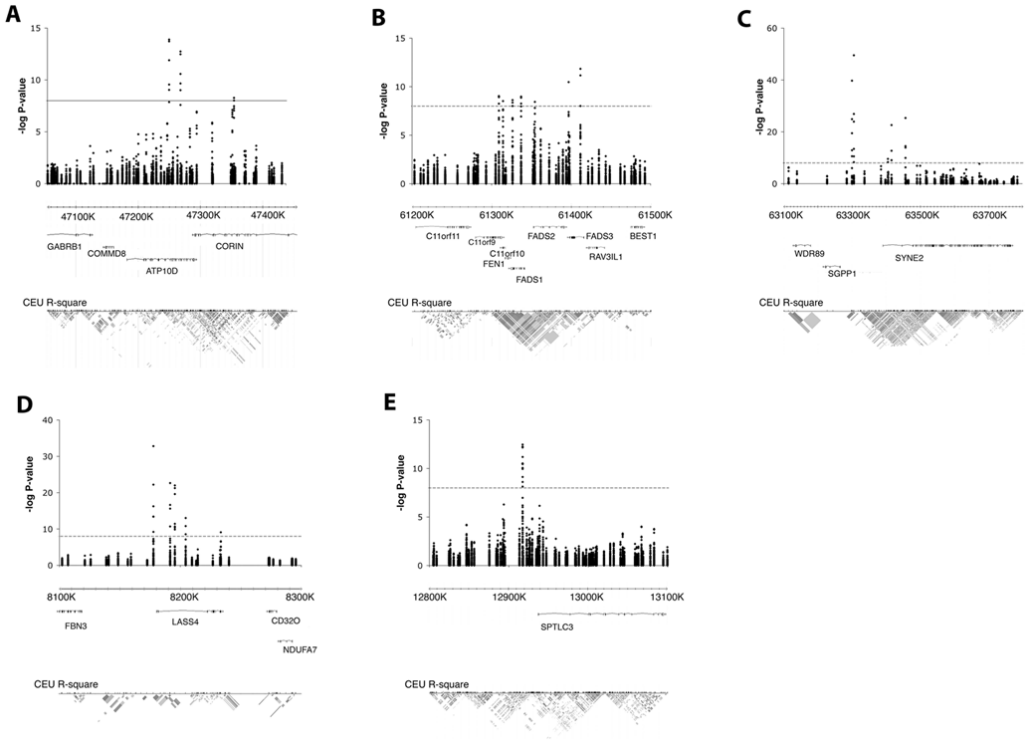
Detailed views of the 5 genomic regions demonstrating significant signals. (A–E) show the 5 regions individually with a representation of all genes near the significant signals and the underlying linkage disequilibrium block structure in the HapMap CEU data (from the UCSC genome browser). Thresholds for significance are indicated by a line.

The GWAS for single species and matched metabolite ratios revealed a total of 32 SNPs in five distinct loci reaching genome-wide significance (*p* values ranging down to 9.08×10^−66^) (Table 1, Figure 1 and Figure 2, and Table S1 and Table S3). The direction and magnitude of the observed effect sizes for the 22 variants identified in the analysis of single species are summarized in Table 1 with full details in Table S1. For three of the regions (chromosomal regions 4p12, 14q23.2 and 19p13.2), *p* values reached genome-wide significance in the largest cohort (South Tyrol), and the effect was replicated in the other populations. For two additional loci (11q12.3 and 20p12.1), signals bordered on genome-wide significance in South Tyrol alone, were consistent between all 5 populations and reached genome-wide significance in the meta-analysis. In the single species analysis, the strongest associations for three of the loci (11q12.3, 14q23.2 and 19p13.2) were found with sphingomyelins and dihydrosphingomyelins. The 4p12 locus showed the strongest association with serum glucosylceramides and the 20p12.1 locus showed the strongest association with serum ceramide concentrations. Table S2 shows the *p*-values for the individual SNPs when included in a multiple regression model, and the fraction of single sphingolipid variance explained by sex, age and all SNPs in the model together. Taken together, the SNPs explain up to 10.1% of the population variation in each trait. Ratios of matched (substrate/product) pairs have been shown to reduce variation in the dataset and increase power of association several orders of magnitude [8]. Analysis of 43 matched metabolite ratios (Table S3) indeed increased power of association up to 10 orders of magnitude on some of the 22 variants above, and revealed an additional 10 SNPs over the same 7 genes reaching statistical significance (see Table S3). Surprisingly no signals from new genes reached genome-wide significance, highlighting the fact that across the 5 cohorts analysed here, the 7 genes identified are the major genes associated with circulating sphingolipid concentrations. Among the 32 significant individual SNPs (Table S4) variants in *LASS4* explain up to 7.5% of the variance in some ratios (i.e. in SM16:0/SM18:0), *SGPP1* variants explain up to 12.7% of the variance (i.e in SM14:0/SM16:0), *FADS1–3* variants explain up to 3.5% of the variance (e.g. in SM16:0/SM16:1), *SPTLC3* variants explain up to 4.9% of the variance (e.g. in SM14:0/SM16:0 and SM24:0/Cer24:0), and *ATP10D* variants up to 4.2% of GluCer/Cer variance. Combined effects of several genes (i.e. *SPTLC3* and *SGPP1*) explains up to 14.2% of the variance in medium chain SM ratios (SM14:0/SM16:0) and, in combination with *LASS4*, up to 11.2% of the variance in long-chain sphingomyelin ratios (SM22:0/SM24:0).

**Table 1:** Variants Significantly Associated with Circulating Sphingolipid Concentrations.

Chr Region	SNP	Effect Allele	Position	Lipid Species With Significant Associations Within the Region	South Tyrol (n = 1097)	Swedish (n = 656)
					P-Value range	P-Value range
4p12 (ATP10D)	rs10938494	A	47258205	GluCer16:0, GluCer24:1, GluCer	**6.7×10^−8^–3.3×10^−13^**	**7.3×10^−2^–4.7×10^−4^**
4p12 (ATP10D)	rs2351791	A	47277144	GluCer16:0, GluCer24:1, GluCer	**2.8×10^−6^–2.9×10^−12^**	**0.039–1.1×10^−4^**
4p12 (ATP10D)	rs4695267	G	47367058	GluCer16:0, GluCer24:1, GluCer	**0.009–5.6×10^−7^**	**0.013–2.0×10^−3^**
11q12.3 (FADS)	rs174537	A	61309256	SM 16:1, 18:1, 20:1	**0.019–3.6×10^−4^**	**0.028–2.7×10^−4^**
11q12.3 (FADS)	rs102275	G	61314379	SM 16:1, 18:1, 20:1	**0.013–2.2×10^−4^**	**0.028–2.6×10^−4^**
11q12.3 (FADS)	rs174546	A	61326406	SM 16:1, 18:1, 20:1	**0.011–2.9×10^−4^**	**0.028–2.7×10^−4^**
11q12.3 (FADS)	rs174556	A	61337211	SM 16:1, 18:1, 20:1	**7.7×10^−3^–8.2×10^−5^**	**0.01–1.9×10^−4^**
11q12.3 (FADS)	rs1535	G	61354548	SM 16:1, 18:1, 20:1	**8.7×10^−3^–6.8×10^−4^**	**0.028–2.1×10^−3^**
11q12.3 (FADS)	rs174449	G	61396955	SM 16:1, 18:1, 20:1	**6.9×10^−3^–3.9×10^−5^**	**0.36–2.2×10^−4^**
11q12.3 (FADS)	rs1000778	A	61411881	SM 16:1, 18:1, 20:1	**5.3×10^−3^–6.3×10^−7^**	**0.11–0.014**
14q23.2 (SGPP1)	rs4902242	G	63299842	SM14:0, 15:0, 23:0, 24:0, 22:1, 24:1, dihSM16:0, 18:0	**0.15–1.7×10^−20^**	**0.35–4.9×10^−10^**
14q23.2 (SGPP1)	rs7157785	A	63305309	SM14:0, 15:0, 23:0, 24:0, 22:1, 24:1, dihSM16:0, 18:0	**0.02–2.5×10^−28^**	**0.79–4.3×10^−11^**
14q23.2 (SGPP1)	rs1959033	A	63405339	SM14:0, 15:0, 23:0, 24:0, 22:1, 24:1, dihSM16:0, 18:0	**0.24–1.8×10^−10^**	**0.97–5.6×10^−3^**
14q23.2 (SGPP1)	rs4459477	A	63415943	SM14:0, 15:0, 23:0, 24:0, 22:1, 24:1, dihSM16:0, 18:0	**0.18–8.1×10^−16^**	**0.47–7.6×10^−6^**
14q23.2 (SGPP1)	rs12889954	G	63457221	SM14:0, 15:0, 23:0, 24:0, 22:1, 24:1, dihSM16:0, 18:0	**0.014–6.1×10^−21^**	**0.54–7.3×10^−4^**
14q23.2 (SGPP1)	rs12881815	A	63674348	SM14:0, 15:0, 23:0, 24:0, 22:1, 24:1, dihSM16:0, 18:0	**0.94–7.6×10^−3^**	**0.90–3.3×10^−7^**
19p13.2 (LASS4)	rs7258249	G	8177721	SM18:0, 18:1, 20:0, 20:1, Cer20:0	**0.056–1.0×10^−15^**	**0.27–8.9×10^−4^**
19p13.2 (LASS4)	rs11666866	A	8191607	SM18:0, 18:1, 20:0, 20:1, Cer20:0	**0.22–7.0×10^−11^**	**0.35–4.0×10^−3^**
19p13.2 (LASS4)	rs1466448	C	8195519	SM18:0, 18:1, 20:0, 20:1, Cer20:0	**0.79–4.2×10^−12^**	**0.037–1.4×10^−5^**
19p13.2 (LASS4)	rs2967625	A	8204411	SM18:0, 18:1, 20:0, 20:1, Cer20:0	**0.65–3.1×10^−9^**	**0.50–9.1×10^−4^**
19p13.2 (LASS4)	rs28133	A	8233502	SM18:0, 18:1, 20:0, 20:1, Cer20:0	**0.051–4.7×10^−5^**	**0.86–3.8×10^−4^**
20p12.1 (SPTLC3)	rs680379	A	12917400	Cer16:0, 22:0, 23:0, 24:0, 24:1, CerSat, CerUnsat, SM17:0, SM16:1	**3.5×10^−4^–1.8×10^−7^**	**0.49–0.035**

Orkney (n = 719)	Croatia (n = 720)	Erf (n = 918)	Joint (n = 4110)	Effect Direction	Effect Direction
P-Value range	P-Value range	P-Value range	P-Value range	Positive Beta	Negative Beta
**0.12–6.3×10^−3^**	**0.12–1.4×10^−3^**	**3.5×10^−4^–5.7×10^−6^**	**6.3×10^−12^–8.0×10^−19^**	GluCer16:0, GluCer24:1, GluCer	
**0.22–0.014**	**0.33–0.01**	**1.2×10^−5^–4.4×10^−8^**	**1.5×10^−11^–4.6×10^−18^**	GluCer16:0, GluCer24:1, GluCer	
**0.18–0.04**	**0.087–0.01**	**3.3×10^−4^–8.1×10^−6^**	**4.9×10^−8^–2.4×10^−12^**	GluCer16:0, GluCer24:1, GluCer	
**0.051–3.3×10^−3^**	**0.24–3.2×10^−4^**	**0.065–1.6×10^−4^**	**2.4×10^−7^–9.0×10^−12^**		SM 16:1, 18:1, 20:1
**0.073–2.8×10^−3^**	**0.19–8.2×10^−4^**	**0.11–4.8×10^−4^**	**3.0×10^−7^–2.2×10^−11^**		SM 16:1, 18:1, 20:1
**0.073–2.8×10^−3^**	**0.18–7.3×10^−4^**	**0.078–1.6×10^−4^**	**2.4×10^−7^–2.0×10^−11^**		SM 16:1, 18:1, 20:1
**9.2×10^−3^–4.9×10^−4^**	**0.28–3.5×10^−4^**	**0.082–5.6×10^−5^**	**8.9×10^−5^–3.7×10^−12^**		SM 16:1, 18:1, 20:1
**0.054–3.9×10^−3^**	**0.28–3.5×10^−4^**	**0.067–7.5×10^−4^**	**4.2×10^−7^–1.6×10^−11^**		SM 16:1, 18:1, 20:1
**0.21–2.9×10^−4^**	**0.41–3.1×10^−4^**	**0.12–0.014**	**3.3×10^−7^–3.0×10^−11^**		SM 16:1, 18:1, 20:1
**0.04–6.2×10^−5^**	**0.05–3.0×10^−3^**	**0.015–4.1×10^−3^**	**6.6×10^−7^–6.6×10^−13^**		SM 16:1, 18:1, 20:1
**0.37–1.5×10^−10^**	**0.93–1.9×10^−13^**	**0.34–8.0×10^−7^**	**0.053–2.3×10^−34^**	SM14:0, 15:0, 22:1	SM23:0, 24:0, 24:1, dihSM16:0, 18:0
**0.66–2.9×10^−15^**	**0.31–5.8×10^−11^**	**0.60–5.2×10^−8^**	**4.9×10^−3^–9.1×10^−66^**	SM14:0, 15:0, 22:1	SM23:0, 24:0, 24:1, dihSM16:0, 18:0
**0.75–8.1×10^−4^**	**0.17–7.8×10^−3^**	**0.76–7.2×10^−3^**	**1.4×10^−3^–6.6×10^−13^**	SM14:0, 15:0, 22:1	SM23:0, 24:0, 24:1, dihSM16:0, 18:0
**0.99–4.7×10^−5^**	**0.69–1.2×10^−7^**	**0.63–6.3×10^−5^**	**0.33–8.3×10^−29^**	SM14:0, 15:0, 22:1	SM23:0, 24:0, 24:1, dihSM16:0, 18:0
**0.69–4.1×10^−6^**	**0.53–6.4×10^−9^**	**0.29–2.4×10^−7^**	**4.1×10^−3^–2.9×10^−33^**	SM14:0, 15:0, 22:1	SM23:0, 24:0, 24:1, dihSM16:0, 18:0
**0.99–0.026**	**0.98–1.7×10^−7^**	**0.73–7.8×10^−3^**	**0.83–7.7×10^−10^**	SM14:0, 15:0, 22:1	SM23:0, 24:0, 24:1, dihSM16:0, 18:0
**0.94–9.6×10^−4^**	**0.001–2.2×10^−7^**	**0.011–9.7×10^−7^**	**1.1×10^−9^–2.3×10^−27^**		SM18:0, 18:1, 20:0, 20:1, Cer20:0
**0.21–1.6×10^−7^**	**0.77–6.3×10^−5^**	**0.68–1.1×10^−3^**	**7.8×10^−6^–6.7×10^−21^**	SM18:0, 18:1, 20:0, 20:1, Cer20:0	
**0.028–7.5×10^−7^**	**0.51–1.3×10^−8^**	**0.67–5.8×10^−4^**	**1.8×10^−5^–4.8×10^−25^**		SM18:0, 18:1, 20:0, 20:1, Cer20:0
**0.48–5.6×10^−4^**	**0.80–0.022**	**0.14–5.2×10^−3^**	**1.2×10^−4^–3.4×10^−15^**	SM18:0, 18:1, 20:0, 20:1, Cer20:0	
**0.31–3.7×10^−5^**	**0.78–1.0×10^−3^**	**0.74–0.053**	**0.031–4.9×10^−11^**	SM18:0, 18:1, 20:0, 20:1, Cer20:0	
**0.10–5.2×10^−5^**	**0.045–3.6×10^−4^**	**0.03–8.3×10^−8^**	**2.2×10^−8^–8.2×10^−15^**	Cer22:0, 23:0, 24:0, 24:1, CerSat, CerUnsat, SM17:0	Cer16:0,SM16:1

22 variants in 7 genes located in 5 distinct chromosomal locations demonstrate genome-wide significant association signals with several measured single sphingolipid species (listed). The *p*-value ranges for significant signals across the sphingolipid species are shown for each population separately and jointly, and the direction of the association effects, as derived from the standardized regression coefficient (β), is summarized. Detailed results for each species along with specific β values are shown in Table S1. Abbreviations sphingomyelin (SM), dihydrosphingomyelin (dihSM), ceramide (Cer) and glucosylceramide (GluCer) unsaturated ceramides (CerUnsat), saturated ceramides (CerSat). In the nomenclature (e.g. SM18:0), the number before the colon refers to length of the carbon chain and the number after the colon to the number of double bonds in the chain. Additional variants uncovered in the matched metabolite ratio analysis can be found in Table S3. Alleles correspond to Illumina TOP notation.

All SNPs within the associated chromosomal regions are located within or are in linkage disequilibrium (LD) with genes that encode enzymes involved in sphingolipid biosynthesis or intracellular transport (Figure 2). The ATPase, class IV, type 10D (*ATP10D*) gene, located at chromosome 4p12, encodes a putative serine-phospholipid (phosphatidylserine, ceramide) translocase [9]. Three SNPs at this locus showed genome-wide significant associations with glucosylceramides (C16:0, C24:1) (Table 1, Table S1), with an additional five variants revealed in the ratio analysis (Table S3). SNP rs10938494 gave the strongest association in the single species analysis (*p*-values of 1.68×10^−9^ in South Tyrol and 8.03×10^−19^ in the joint analysis), and was among the strongest association in the ratio analysis (*p* = 3.04×10^−16^) along with rs2351791 (*p* = 6.58×10^−17^).

Three fatty-acid desaturase genes (*FADS1*, *2* and *3*) are located adjacent to one another in a cluster at the 11q12.3 locus. The *FADS1–3* genes encode enzymes that regulate the desaturation of fatty acids by the introduction of double bonds between defined carbons of the fatty acyl chain. Seven SNPs at this locus, distributed in and around the three genes, reached statistical significance in the single species analysis for sphingomyelin 16∶1 levels in the joint analysis, with *p*-values ranging from 2.99×10^−11^ (rs174449, close to *FADS3*) to 6.60×10^−13^ (rs1000778, in *FADS3*) (Table 1). The ratio analysis revealed an additional SNP at this locus within the *FADS3* gene (rs174450, Table S3), and improved association results for other SNPs several orders of magnitude (e.g. rs1000778 *p* = 1.29×10^−15^). Fatty acids are built into ceramides by the ceramide synthases (e.g. *LASS4*). Unsaturated ceramides can be synthesized exclusively by the introduction of unsaturated fatty acids into the sphingosine/sphinganine chain. The pivotal role of *FADS1*–*3* in the synthesis of unsaturated ceramides is confirmed by the strong associations of SNPs in this cluster to the mono-unsaturated sphingomyelins 16∶1, 18∶1 and 20∶1, which are the end-products of the ceramide biosynthesis pathway (Table 1, Table S1), and the ratios between these and their respective unsaturated precursors (Table S3). Previous studies of sphingolipid metabolites and poly-unsaturated fatty acids (PUFA) have demonstrated associations to SNPs, including rs174537, over the *FADS1* and *FADS2* genes in several populations [8],[10],[11].

**Figure 3:**
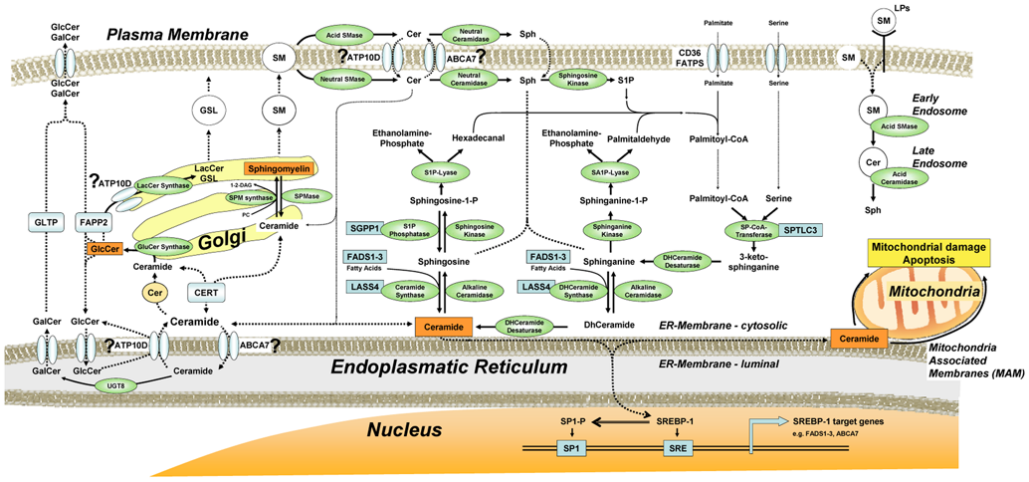
Major sphingolipid synthesis and trafficking pathways. Biosynthetic pathways are shown along with the position in these pathways of enzymes encoded by the genes giving statistically significant associations for circulating sphingolipid concentrations.

The sphingosine-1-phosphate phosphohydrolase 1 gene (*SGPP1*) at the 14q23.2 locus belongs to the super-family of lipid phosphatases that catalyze the generation of sphingosine and, together with irreversible cleavage by sphingosine-1-phosphate (S1P)-lyase, strongly influences the pathway of S1P to ceramide (Figure 3). Six SNPs in and around this gene demonstrate the most significant associations with circulating sphingomyelin C14–C16/C22–C24 and dihydrosphingomyelin concentrations (Table 1) in the single species analysis, with a further two SNPs revealed in the ratio analysis. SNP rs7157785 showed the strongest association with sphingomyelin 14∶0 relative content (molar percentage: mol%) with genome-wide significant *p*-values in all five populations, particularly in the South Tyrol population (*p* = 2.53×10^−28^) and joint analysis (*p* = 9.08×10^−66^), and demonstrated the most significant association in the ratio analysis. Enhanced SGPP1 activity leads to elevated ceramide levels by shifting the stochiometric balance of SGPP1/S1P-lyase towards sphingosine and ceramide production.

Five SNPs at the 19p13.2 locus showed some of the strongest associations with sphingolipids and all lie within *LASS4*, the gene encoding LAG1 longevity assurance homologue 4. In the single species analysis SNP rs7258249 showed the highest genome-wide significant association with sphingomyelin 18∶0 mol% (South Tyrol *p* = 1.04×10^−15^ and joint analysis *p* = 2.28×10^−27^). Several *LASS4* SNPs showed statistically significant association with the sphingomyelin species C18 to C20 and with ceramide C20∶0 (Table 1 and Table S1). In the ratio analysis, however, associations strengthened by several orders of magnitude (*p* value) over those with these SNPs, with rs1466448 demonstrating the most statistically significant association (*p* = 4.05×10^−35^). LASS family members, six of which have been identified in mammals (*LASS1–6*), are *de novo* ceramide synthases (CerS) that synthesize dihydroceramide from sphinganine and fatty acid (Figure 3). Moreover, LASS enzymes catalyze the re-synthesis of ceramide and phytoceramide from sphingosine and phytosphingosine respectively, which are cleavage products of alkaline ceramidase activity in endoplasmic reticulum (ER) membranes.

The 20p12.1 locus contains the serine palmitoyltransferase long chain base subunit 3 gene (*SPTLC3*) encoding a functional subunit of the SPT enzyme-complex that catalyzes the first and rate-limiting step of *de novo* sphingolipid synthesis. One SNP (rs680379) demonstrated association for unsaturated ceramide in the South Tyrol population alone (*p* = 1.77×10^−07^) and was genome-wide significant in the joint analysis (*p* = 8.24×10^−15^). Significant association was observed also with C16 to C24 ceramides and the sphingomyelins 16∶1 and 17∶0 (Table 1 and Table S1). The ratio analysis strengthened association at this variant (*p* = 3.3×10^−20^ for the metabolite ratio SM24:0/Cer24:0) and revealed two further significant variants at this locus (rs3848751 and rs6078866, Table S3).

As matched metabolite ratios can serve as a proxy for enzyme activity [8], in a complementary candidate gene approach, we investigated association signals in our combined single species and ratio datasets at 624 SNPs within or near 40 genes that encode enzymes involved in sphingolipid metabolism, in order to identify the most promising variants within these genes for further analysis. Of these, a total of 70 variants in or near 23 of the genes demonstrate association *p* values of 10^−4^ or less (Table S5).

Sex and age adjusted single sphingolipids species displayed strong phenotypic correlations with circulating plasma lipoproteins especially with total cholesterol or LDL-cholesterol (Table S6, e.g. between the sum of saturated sphingomyelin species and total cholesterol: 0.788/0.717/0.794/0.733/0.773 in respectively NPHS/ERF/SOUTH TYROL/CROATIA/ORKNEY; or SM16:1 and total cholesterol 0.737/0.631/0.671/0.6/0.638). This is in agreement with recent lipid profiling of lipoprotein fractions, showing higher proportions of sphingomyelin and ceramides in the LDL fraction [12]. However, among the GWAS hits uncovered in this analysis, only the *FADS1–3* cluster overlaps with those reported in large meta-analysis of circulating serum lipoproteins levels (strongest with total and LDL-cholesterol levels) [13]. Several of the variants reported here display suggestive associations with classical lipids in the EUROSPAN cohorts (Table S7). All eight SNPs in the *FADS1–3* cluster associate with HDL-cholesterol levels (age-sex adjusted *p* values between 0.06 and 0.0041) similar to previous observations [8]. Interestingly, the sex-specific age-adjusted results show that these associations seem driven by the association found in males (lowest *p* = 0.0037 at rs174546). Association with HDL-cholesterol in males is also seen with SNPs in *ATP10D* (rs2351791, *p* = 0.01) and *SPTLC3* (rs3848751, *p* = 0.0047). SNPs at *ATP10D* also associate with LDL-cholesterol, albeit weakly in the total population (rs469463, *p* = 0.034). In the males only, variants at *LASS4* (rs28133, *p* = 0.043) and *SPTLC3* (rs3848751, *p* = 0.022 and rs6078866, *p* = 0.02) also associate weakly with LDL-cholesterol levels. Five variants in *FADS1–3* and two in *ATP10D* associate with triglyceride levels, with lower *p* values in males than in the whole group (*p* values from 0.017 to 0.009 in *FADS1–3* and 0.0071 for rs17462424 in *ATP10D*). Association of *FADS* variants with triglyceride levels has also been observed in other populations [8]. As previously highlighted [8], the *p* values for association with the sphingolipids species were orders of magnitude stronger than with these classical lipids.

Given the reported associations to classical lipids and cardiovascular disease with variants at the *FADS1–3* locus [10],[13],[14], and the evidence from functional studies of a role for sphingolipids in atherosclerotic plaque formation and lipotoxic cardiomyopathy [15], we looked *in silico* in a series of three age- and sex-adjusted GWAS datasets of German myocardial infarction (MI) case-control studies (Ger MIFS I [16] Ger MIFS II [17] and Ger MIFS III (KORA), unpublished) for evidence of association with the major variants associating with sphingolipid concentrations. Variants within three of the genes (*ATP10D*, *FADS3* and *SPTLC3*) associate with MI in one or more of the studies (Table 2). The protective odds ratios observed for variants in *ATP10D* and *SPTLC3* are on alleles correlating positively with higher metabolite/lower ceramide ratios (i.e. GluCer/Cer and SM/Cer), in support of evidence that increased enzyme/transporter activity that lowers ceramide levels might alleviate the pro-apoptotic effects seen with higher ceramide levels in cardiomyocytes [18]. As previously hypothesised, carriers of FADS variants that are associated with higher desaturase activity may be prone to a proinflammatory response favoring atherosclerotic vascular damage [14].

**Table 2:** Association of Variants Influencing Sphingolipid Concentrations with MI in 3 German Studies.

RS_ID	CHR	GENE	BP	A1	A2	P gwasI (N = 2503)	OR (95CI) I	P gwasII (N = 2506)	OR (95CI) II	P gwasIII (N = 2597)	OR (95CI) III	Combined P	Meta OR (95CI)	Significant difference from 1
rs4298115	4	ATP10D	47255143	T	C	**0.0109**	1.20 (1.04–1.39)	0.4541	1.05 (0.93–1.18)	0.3064	1.06 (0.95–1.18)	**0.0180**	**1.08 (1.01–1.17)**	**yes**
rs10938494	4	ATP10D	47258205	A	G	**7.25E-03**	0.8 (0.67–0.94)	0.1066	0.89 (0.77–1.03)	**0.0632**	0.88 (0.77–1.01)	**5.13E-04**	**0.86 (0.79–0.94)**	**yes**
rs2351791	4	ATP10D	47277144	A	C	**0.0260**	0.83(0.70–0.98)	0.2676	0.93 (0.81–1.06)	**0.0325**	0.87 (0.77–0.99)	**1.83E-03**	**0.87 (0.80–0.95)**	**yes**
rs17462424	4	ATP10D	47293055	C	T	**2.05E-03**	1.26 (1.09–1.46)	0.3631	1.06 (0.94–1.2)	0.3395	1.06 (0.94–1.18)	**7.44E-03**	**1.10 (1.02–1.19)**	**yes**
rs6832495	4	ATP10D	47304421	G	A	0.1312	0.90 (0.78–1.03)	0.1693	0.92 (0.81–1.04)	0.4592	0.96 (0.86–1.07)	**0.0430**	**0.93 (0.87–0.99)**	**yes**
rs4694863	4	ATP10D	47330343	C	A	**1.89E-03**	1.28 (1.1–1.49)	0.3236	1.07 (0.94–1.22)	0.8476	1.01 (0.90–1.15)	**0.0237**	**1.09 (1.01–1.18)**	**yes**
rs2351784	4	ATP10D	47364192	C	T	0.1681	1.10 (0.96–1.27)	0.2570	1.07 (0.95–1.21)	0.9717	0.99 (0.90–1.11)	0.1856	1.05 (0.98–1.12)	no
rs174537	11	FADS1	61309256	T	G	0.9914	1.0 (0.86–1.16)	0.6780	0.97 (0.86–1.11)	0.1614	1.09 (0.97–1.23)	0.5200	1.02 (0.95–1.11)	no
rs102275	11	FADS1	61314379	C	T	0.9914	1.0 (0.86–1.16)	0.6780	0.97 (0.86–1.11)	0.1381	1.09 (0.97–1.23)	0.4896	1.02 (0.95–1.11)	no
rs174546	11	FADS1	61326406	T	C	0.9660	1.00 (0.86–1.17)	0.6684	0.97 (0.86–1.11)	0.1335	1.10 (0.97–1.23)	0.4725	1.03 (0.95–1.11)	no
rs174556	11	FADS1	61337211	T	C	0.8739	1.01 (0.87–1.19)	0.8321	0.99 (0.86–1.13)	0.2444	1.08 (0.95–1.22)	0.4879	1.03 (0.95–1.11)	no
rs1535	11	FADS2	61354548	G	A	0.9920	1.00 (0.86–1.16)	0.8636	0.99 (0.87–1.12)	0.1198	1.10 (0.98–1.24)	0.3710	1.03 (0.96–1.11)	no
rs174450	11	FADS3	61398118	G	T	**2.49E-03**	0.80 (0.70–0.93)	**0.0129**	0.86 (0.76–0.97)	0.8737	0.99 (0.88–1.11)	**2.01E-03**	**0.89 (0.83–0.96)**	**yes**
rs1000778	11	FADS3	61411881	A	G	**0.0193**	0.82 (0.69–0.97)	0.5120	1.05 (0.91–1.20)	0.6491	0.97 (0.85–1.10)	0.2932	0.96 (0.88–1.04)	no
rs11158515	14	SGPP1	63385073	G	T	0.2460	0.92 (0.8–1.06)	0.9837	1.00 (0.89–1.13)	0.1260	0.92 (0.82–1.02)	0.1223	0.95 (0.88–1.01)	no
rs1959033	14	SGPP1	63405339	A	G	0.1685	0.82 (0.62–1.09)	0.9757	0.99 (0.79–1.26)	0.6485	1.05 (0.85–1.30)	0.6984	0.97 (0.85–1.12)	no
rs4459477	14	SGPP1	63415943	T	C	0.3857	1.12 (0.87–1.44)	0.9151	0.99 (0.82–1.20)	**0.0143**	0.79 (0.67–0.95)	0.2321	0.93 (0.83–1.04)	no
rs12889954	14	SGPP1	63457221	C	T	0.8460	0.98 (0.81–1.19)	0.8786	1.01 (0.86–1.19)	0.5553	1.05 (0.90–1.22)	0.7128	1.02 (0.93–1.12)	no
rs12881815	14	SGPP1	63674348	A	G	0.3616	1.16 (0.85–1.59)	0.7387	1.05 (0.80–1.37)	0.3404	1.13 (0.88–1.46)	0.2106	1.11 (0.94–1.29)	no
rs3848751	20	SPTLC3	12913401	G	T	**8.04E-03**	0.81 (0.70–0.95)	0.6096	0.97 (0.85–1.10)	**0.0397**	0.88 (0.79–0.99)	**3.54E-03**	**0.89 (0.82–0.96)**	**yes**
rs6078866	20	SPTLC3	12922567	G	A	**8.04E-03**	0.81 (0.70–0.95)	0.5377	0.96 (0.85–1.09)	**0.0376**	0.88 (0.79–0.99)	**2.75E-03**	**0.89 (0.83–0.96)**	**yes**

Association signals with 21 (from 32) variants in 4 chromosomal locations showing genome-wide significant association to circulating sphingolipids, with MI in 3 distinct German patient studies, GerMIFS-I, -II and –III (KORA), differing in their composition by family history of MI [16],[17]. 11 variants across the 5 genes (including all *LASS4* variants) were removed due to low imputation quality (Rsq<0 .7) in at least one of the MI cohorts or the control groups (KORAS3, F4 and/or PopGen). Reported *p* values are age and sex adjusted. A fixed-effects meta-analysis using inverse-variance weighting was used to derive combined odds ratios (Meta OR).

## Discussion

Direct experimental evidence indicates a role for sphingolipids in several common complex chronic disease processes including atherosclerotic plaque formation, myocardial infarction (MI), cardiomyopathy, pancreatic beta cell failure, insulin resistance and type 2 diabetes mellitus (T2D) [15]. Until now, the genetic variants that influence circulating sphingolipid concentrations in the general population have been characterized in relatively small cohorts [8]. Here we identified genetic variation with a significant effect on the biosynthesis, metabolism or intracellular trafficking of some of the major sphingolipids species in a large diverse group of European population samples. The SNPs showing association with circulating sphingolipids explain up to 10.1% of the population variation in each trait and 14.2% of some matched ratios (Tables S2 and Table S4). Four of the five loci identified contain genes encoding proteins that are either responsible for *de novo* ceramide synthesis or for ceramide re-synthesis from sphingosine/sphinganine-phosphates or both (*SPTLC3*, *LASS4*, *FADS1–3* and *SGPP1*). Increases in all of these enzymatic activities are predicted to elevate the “ceramide-pool”. The associations are observed not only with ceramides, but also with sphingomyelins, indicating that a considerable proportion of ceramide is converted into the large and more stable “sphingomyelin-pool”. None of the genes involved in ceramide degradation or ceramide-related signaling is genome-wide significantly associated with the traits analyzed, indicating the primary role of genes related to ceramide production in the genetic control of ceramide levels. Of these four loci, the *FADS1–3* gene cluster has been the most frequently to be reported linked with disease in recent literature. Variants within in this region have been associated with cardiovascular disease and classic lipid risk factors such as cholesterol levels [10],[13],[14]. Reported variants demonstrating association in these reports (rs174547, rs174570, rs174537 and rs174546) are within the *FADS1* and *FADS2* genes, but expression studies indicate complex regulation in this region, with the *FADS1* SNP rs174547 showing correlation with expression of both *FADS1* and *FADS3* genes [19], while the *FADS1* SNP rs174546 correlates with *FADS1* but not *FADS2* expression [10]. Our strongest associations with both sphingolipid levels and MI are in or nearest the *FADS3* gene, with variants showing less marked association with cholesterol levels than that observed with variants over *FADS1* and *FADS2* genes (Table S7). It is known that sphingomyelin and ceramides can modulate the atherogenic potential of LDL [20]. Further functional studies will be necessary to determine whether the active mechanism is through *FADS3* alone, or in concert with *FADS1*, *FADS2* or both.

Neurological phenotypes associated with *FADS2* include attention-deficit/hyperactivity disorder [21] and the moderation of breastfeeding effects on IQ [22]. Little is published regarding disease association with variants at the other four major loci described here. However, a reported association between expression levels of *SGPP1* with Schizophrenia [23] along with changes in *SPTLC2* (with variants identified in our candidate SNP search –Table S4) and *ASAH1*, highlights the importance of testing variants in these genes with multiple neurological and psychiatric diseases. Additional neurological associations with candidate genes listed in Table S4 include *SGPL1* in Alzheimer's disease [24] and GBA with Parkinson's disease and dementia with Lewy bodies [25],[26]. The wider possible involvement of genes within pathways of ceramide metabolism in Lewy body disease has also been recently reviewed [27].

The fifth locus contains *ATP10D*, a cation transport ATPase (P-type) type IV subfamily member. The type IV subfamily is thought to be an important regulator of intracellular serine-phospholipid trafficking however the exact function or transport specificity of *ATP10D* has not yet been described [9]. A novel functional finding of this study is the specificity of the association of *ATP10D* SNPs to glucosylceramides (among the species tested so far), which provides the first evidence for the functional involvement of *ATP10D* in intracellular transport of specific species of ceramide (Figure 3). Impaired function of *ATP10D* may therefore lead to enhanced exposure of ceramide to glucosyltransferases, forming higher concentrations of glycosylceramides that are released into the plasma compartment or may elevate serum glucosylceramide concentrations by impaired transport of glycosylceramide to the trans Golgi network. Mutations of *ATP10D* (C57BL/6J(B6)) in mice result in low HDL concentrations and these mice develop severe obesity, hyperglycaemia and hyperinsulinaemia when fed on a high-fat diet [28]. Based on the mouse model, increased circulating glucosylceramides in connection with *ATP10D* function would be one plausible mechanism of contributing to weight gain and early insulin resistance. From the novel association of SNPs in *ATP10D* to MI (Table 2) seen in German studies, further investigation of the specific role of glucosylceramides in MI and other cardiovascular diseases is warranted.

Thus, sphingolipids play a role in pathological processes leading to common complex diseases, and identification of genetic variants that influence the balance between individual sphingolipid species is an important first step into dissecting out the genetic components in such processes. Associations between the SNPs identified in this study, some of which have a strong effect on the circulating plasma levels, and complex metabolic, cardiovascular, inflammatory and neurological diseases in which a role for a sphingolipid-dependent mechanism is implicated can now be investigated. Modulation of sphingolipids *in vivo* has demonstrated that this may be a successful preventative strategy for diseases in which sphingolipids play a role, lending hope that, once such disease contributions are identified, successful therapeutic regimes may subsequently be identified.

## Materials and Methods

### Ethics statement

All studies were approved by the appropriate Research Ethics Committees. The Northern Swedish Population Health Study (NSPHS) was approved by the local ethics committee at the University of Uppsala (Regionala Etikprövningsnämnden, Uppsala). The ORCADES study was approved by the NHS Orkney Research Ethics Committee and the North of Scotland REC. The Vis study was approved by the ethics committee of the medical faculty in Zagreb and the Multi-Centre Research Ethics Committee for Scotland. The ERF study was approved by the Erasmus institutional medical-ethics committee in Rotterdam, The Netherlands. The MICROS study was approved by the ethical committee of the Autonomous Province of Bolzano. For the German MI studies (GerMIFS-I,-II and –III(KORA), local ethics committees approved the studies and written informed conset obtained as published previously.

### Study populations

The ERF study is a family-based study which includes over 3000 participants descending from 22 couples living in the Rucphen region in the 19th century. All descendants were invited to visit the clinical research center in the region where they were examined in person and where blood was drawn (fasting). Height and weight were measured for each participant. All participants filled out questionnaire on risk factors, including smoking. The 800 participants included in the lipidomics studies consisted of the first series of participants.

The MICROS study is part of the genomic health care program ‘GenNova’ and was carried out in three villages of the Val Venosta on the populations of Stelvio, Vallelunga and Martello. This study was an extensive survey carried out in South Tyrol (Italy) in the period 2001–2003. An extensive description of the study is available elsewhere [29]. Briefly, study participants were volunteers from three isolated villages located in the Italian Alps, in a German-speaking region bordering with Austria and Switzerland. Due to geographical, historical and political reasons, the entire region experienced a prolonged period of isolation from surrounding populations. Information on the health status of participants was collected through a standardized questionnaire. Laboratory data were obtained from standard blood analyses. Genotyping was performed on just under 1400 participants with 1334 available for analysis after data cleaning. All participants were included in the lipidomics studies.

The Swedish samples are part of the Northern Swedish Population Health Study (NSPHS) representing a family-based population study including a comprehensive health investigation and collection of data on family structure, lifestyle, diet, medical history and samples for laboratory analyses. Samples were collected from the northern part of the Swedish mountain region (County of Norrbotten, Parish of Karesuando). Historic population accounts show that there has been little immigration or other dramatic population changes in this area during the last 200 years.

The Orkney Complex Disease Study (ORCADES) is an ongoing family-based, cross-sectional study in the isolated Scottish archipelago of Orkney. Genetic diversity in this population is decreased compared to Mainland Scotland, consistent with the high levels of endogamy historically. Data for participants aged 18 to 100 years, from a subgroup of ten islands, were used for this analysis. Fasting blood samples were collected and over 200 health-related phenotypes and environmental exposures were measured in each individual. All participants gave informed consent and the study was approved by Research Ethics Committees in Orkney and Aberdeen.

The Vis study includes a 986 unselected Croatians, aged 18–93 years, who were recruited into the study during 2003 and 2004 from the villages of Vis and Komiza on the Dalmatian island of Vis [30],[31]. The settlements on Vis island have unique population histories and have preserved their isolation from other villages and from the outside world for centuries. Participants were phenotyped for 450 disease-related quantitative traits. Biochemical and physiological measurements were performed, detailed genealogies reconstructed, questionnaire of lifestyle and environmental exposures collected, and blood samples and lymphocytes extracted and stored for further analyses. Samples in all studies were taken in the fasting state.

### Lipidomics

Lipids were quantified by electrospray ionization tandem mass spectrometry (ESI-MS/MS) in positive ion mode as described previously [32],[33]. EDTA plasma (serum for South Tyrol) samples were quantified upon lipid extraction by direct flow injection analysis using the analytical setup described by Liebisch et al. [33]. A precursor ion scan of m/z 184 specific for phosphocholine containing lipids was used for phosphatidylcholine (PC) and sphingomyelin (SM) [33]. Ceramide and hexosylceramide were analyzed using a fragment ion of m/z 264 [32]. For each lipid class two non-naturally occurring internal standards were added and quantification was achieved by calibration lines generated by addition of naturally occurring lipid species to plasma. Deisotoping and data analysis for all lipid classes was performed by self programmed Excel Macros according to the principles described previously [33]. Nomenclature of sphingomyelin species is based on the assumption that d18∶1 (dihydroxy 18∶1 sphingosine) is the main base of plasma sphingomyelin species, where the first number refers to the number of carbon atoms in the chain and the second number to the number of double bonds in the chain.

### Genotyping

DNA samples were genotyped according to the manufacturer's instructions on Illumina Infinium HumanHap300v2 (except for samples from Vis for which version 1 was used) or HumanCNV370v1 SNP bead microarrays. Four populations have 318,237 SNP markers in common that are distributed across the human genome, with Vis samples having 311,398 SNPs in common with the other populations. Samples with a call rate below 97% were excluded from the analysis. Sphingolipid measurements were available for analysis following quality control assessment for 4110 study participants.

### Statistical analysis

Genome-wide association analysis was performed using the GenABEL package in R [34]. A score test was used to test for association between the age- and sex-adjusted residuals of sphingolipid traits (both as absolute concentrations and as relative content of the total sphingolipid pool: mol%) and SNP genotypes using an additive model. The Genomic Control procedure [35] was used to account for under-estimation of the standard errors of effects, which occurs because of pedigree structure present in the data [36]. For the most interesting results and the species ratios, we re-analysed the data using “mmscore” function, a score test for family-based association [37], as implemented in GenABEL. The relationship matrix used in analysis was estimated using genomic data with “ibs” (option weight = “freq”) function of GenABEL. This analysis, accounting for pedigree structure in an exact manner, allowed for unbiased estimation of the effects of the genetic variants (adjusted for age and sex). The results from all cohorts were combined into a fixed-effects meta-analysis with reciprocal weighting on standard errors of the effect-size estimates, using MetABEL (http://mga.bionet.nsc.ru/~yurii/ABEL/). Thresholds for genome wide significance were set at a *p* value of less than 1.57×10^−7^ (0.05/318,237 SNPs) for the individual populations. For the overall meta-analysis we chose to use the conservative threshold of 7.2×10^−8^ [38]. Since many of the traits tested and especially the ratios demonstrate high degrees of correlation, introducing a suitable statistical correction the multiple testing of the 76 correlated traits would be complex. Since Bonferroni correction (unsuitable in this instance) would lower thresholds to values between *p* = 10^−9^ to 10^−10^, and since all five genomic regions have variants with *p* values <10^−10^, we report the age-sex corrected *p* values alone. The threshold for replication of significant results from one population in other cohorts was set at a *p*-value less than 0.05 divided by the number of SNPs tested. All significant variants reported are in Hardy-Weinberg Equilibrium, and effect directions are consistent across all five populations.

## Supporting Information

Table S1 Variants significantly associated with circulating sphingolipid concentrations. 22 variants in 5 distinct chromosomal locations demonstrate genome-wide significant association signals with several measured sphingolipid species (listed). The *p*-values for significant signals across the sphingolipid species are shown for each population separately and jointly, and the direction of the association effects, as derived from the standardized regression coefficient (β), is provided. Abbreviations, sphingomyelin (SM), dihydrosphingomyelin (dihSM), ceramide (Cer) and glucosylceramide (GluCer) unsaturated ceramides (CerUnsat), saturated ceramides (CerSat). In the nomenclature (e.g. GluCer18:0), the number before the colon refers to length of the carbon chain and the number after the colon to the number of double bonds in the chain. Where mol% is used, the measure refers to the relative content of the measured species in the total sphingolipid pool, and is independent of other associated lipid species. Sex-specific age adjusted analyses provided little additional information, unlike the case of the ratio analyses (see Table S3), and is not shown.
(0.28 MB XLS)

Table S2 Variance in circulating sphingolipid concentrations. The upper part of the table shows *p*-values (NS - not significant *p*-value >0.05) estimated using a multiple regression model. The bottom part of the table, shows the fraction of variance of the traits explained by sex, age and all the significant SNPs from the regression model.
(0.06 MB XLS)

Table S3 Variants significantly associated with matched metabolite sphingolipid ratios. 32 variants in 5 distinct chromosomal locations demonstrate genome-wide significant association signals with matched metabolite ratios designed to probe metabolism (11 ratios), desaturation (16 ratios) and elongation (16 ratios) - details of the ratios are provided in the table. The *p*-values for significant signals across the sphingolipid species are shown for each population separately and jointly, and the direction of the association effects, as derived from the standardized regression coefficient (β), is provided. Sex-specific age adjusted results are also displayed, as these provided additional information with the ratio analysis that was more significant than the sex-specific effects seen in the analysis of the single species (not shown).
(0.09 MB XLS)

Table S4 Proportion of variance in matched shpingolipid metabolite ratios. Proportion of the variance in age and sex adjusted sphingolipid ratio explained by SNP variants that were significant in the GWA metanalysis of the 5 EUROSPAN populations. General linear mixed models were fitted using the polygenic function of the R statistical package “GenABEL” and variances explained drawn from comparing residual variances between models fitting in the SNP tested as fixed effects and models not fitting them in. Single SNP analysis were carried out for all candidate SNP, and multiple SNP for traits influenced by multiple candidate regions (in this case the top SNP for each region was selected). Shaded cells indicate SNP with GWA significant association in the meta-analysis for the trait analysed.
(0.03 MB XLS)

Table S5 Signals over SNPs within candidate sphingolipid genes. Using a dataset of 624 SNPs within or near 40 genes encoding enzymes and transporters involved in pathways of sphingolipid metabolism, association results were extracted from both the single sphingolipid GWAS runs, or those with the matched metabolite ratios. In total 70 variants within and around 23 of these genes demonstrate p values of 10^−4^ or less, making them interesting targets for further study.
(0.09 MB XLS)

Table S6 Table of phenotypic correlations between traits. Pearson correlations of age and sex adjusted measures were calculated and only significant values (2 tailed *p*-values < = 0.05) represented. Traits included all sphingolipids species, some anthropometric measures: weight, bmi and height, blood pressure (sbp = systolic blood pressure, dbp = diastolic blood pressure),and classical circulating lipoproteins species tc = total cholesterol, ldl = LDL cholesterol, hdl = HDL-cholesterol, tri = Triglycerides.
(0.40 MB XLS)

Table S7 Association signals for sphingolipid SNPs with classical lipids. Signals were extracted from age-sex adjusted or age adjusted sex specific GWAS scans across the EUROSPAN populations for the traits: HDL- and LDL-cholesterol, Triglycerides (tri) and Total Cholesterol (tc).
(0.26 MB XLS)

## References

[1] Pruett ST, Bushnev A, Hagedorn K, Adiga M, Haynes CA (2008). Sphingolipids. Biodiversity of sphingoid bases (“sphingosines”) and related amino alcohols.. J Lipid Res.

[2] Zheng W, Kollmeyer J, Symolon H, Momin A, Munter E (2006). Ceramides and other bioactive sphingolipid backbones in health and disease: lipidomic analysis, metabolism and roles in membrane structure, dynamics, signaling and autophagy.. Biochim Biophys Acta.

[3] Kolter T, Sandhoff K (2006). Sphingolipid metabolism diseases.. Biochim Biophys Acta.

[4] Dawkins JL, Hulme DJ, Brahmbhatt SB, Auer-Grumbach M, Nicholson GA (2001). Mutations in SPTLC1, encoding serine palmitoyltransferase, long chain base subunit-1, cause hereditary sensory neuropathy type I.. Nat Genet.

[5] Simpson MA, Cross H, Proukakis C, Priestman DA, Neville DC (2004). Infantile-onset symptomatic epilepsy syndrome caused by a homozygous loss-of-function mutation of GM3 synthase.. Nat Genet.

[6] Schulz A, Mousallem T, Venkataramani M, Persaud-Sawin DA, Zucker A (2006). The CLN9 protein, a regulator of dihydroceramide synthase.. J Biol Chem.

[7] Wang E, Norred WP, Bacon CW, Riley RT, Merrill AHJ (1991). Inhibition of sphingolipid biosynthesis by fumonisins. Implications for diseases associated with Fusarium moniliforme.. J Biol Chem.

[8] Gieger C, Geistlinger L, Altmaier E, Hrabe de Angelis M, Kronenberg F (2008). Genetics meets metabolomics: a genome-wide association study of metabolite profiles in human serum.. PLoS Genet.

[9] Flamant S, Pescher P, Lemercier B, Clement-Ziza M, Kepes F (2003). Characterization of a putative type IV aminophospholipid transporter P-type ATPase.. Mamm Genome.

[10] Tanaka T, Shen J, Abecasis GR, Kisialiou A, Ordovas JM (2009). Genome-wide association study of plasma polyunsaturated fatty acids in the InCHIANTI Study.. PLoS Genet.

[11] Schaeffer L, Gohlke H, Muller M, Heid IM, Palmer LJ (2006). Common genetic variants of the FADS1 FADS2 gene cluster and their reconstructed haplotypes are associated with the fatty acid composition in phospholipids.. Hum Mol Genet.

[12] Wiesner P, Leidl K, Boettcher A, Schmitz G, Liebisch G (2009). Lipid profiling of FPLC-separated lipoprotein fractions by electrospray ionization tandem mass spectrometry.. J Lipid Res.

[13] Aulchenko YS, Ripatti S, Lindqvist I, Boomsma D, Heid IM (2009). Loci influencing lipid levels and coronary heart disease risk in 16 European population cohorts.. Nat Genet.

[14] Martinelli N, Girelli D, Malerba G, Guarini P, Illig T (2008). FADS genotypes and desaturase activity estimated by the ratio of arachidonic acid to linoleic acid are associated with inflammation and coronary artery disease.. Am J Clin Nutr.

[15] Holland WL, Summers SA (2008). Sphingolipids, insulin resistance, and metabolic disease: new insights from in vivo manipulation of sphingolipid metabolism.. Endocr Rev.

[16] Samani NJ, Erdmann J, Hall AS, Hengstenberg C, Mangino M (2007). Genomewide association analysis of coronary artery disease.. N Engl J Med.

[17] Erdmann J, Grosshennig A, Braund PS, Konig IR, Hengstenberg C (2009). New susceptibility locus for coronary artery disease on chromosome 3q22.3.. Nat Genet.

[18] Bielawska AE, Shapiro JP, Jiang L, Melkonyan HS, Piot C (1997). Ceramide is involved in triggering of cardiomyocyte apoptosis induced by ischemia and reperfusion.. Am J Pathol.

[19] Kathiresan S, Willer CJ, Peloso GM, Demissie S, Musunuru K (2009). Common variants at 30 loci contribute to polygenic dyslipidemia.. Nat Genet.

[20] Schissel SL, Jiang X, Tweedie-Hardman J, Jeong T, Camejo EH (1998). Secretory sphingomyelinase, a product of the acid sphingomyelinase gene, can hydrolyze atherogenic lipoproteins at neutral pH. Implications for atherosclerotic lesion development.. J Biol Chem.

[21] Brookes KJ, Chen W, Xu X, Taylor E, Asherson P (2006). Association of fatty acid desaturase genes with attention-deficit/hyperactivity disorder.. Biol Psychiatry.

[22] Caspi A, Williams B, Kim-Cohen J, Craig IW, Milne BJ (2007). Moderation of breastfeeding effects on the IQ by genetic variation in fatty acid metabolism.. Proc Natl Acad Sci U S A.

[23] Narayan S, Head SR, Gilmartin TJ, Dean B, Thomas EA (2009). Evidence for disruption of sphingolipid metabolism in schizophrenia.. J Neurosci Res.

[24] Morgan AR, Turic D, Jehu L, Hamilton G, Hollingworth P (2007). Association studies of 23 positional/functional candidate genes on chromosome 10 in late-onset Alzheimer's disease.. Am J Med Genet B Neuropsychiatr Genet.

[25] Nichols WC, Pankratz N, Marek DK, Pauciulo MW, Elsaesser VE (2009). Mutations in GBA are associated with familial Parkinson disease susceptibility and age at onset.. Neurology.

[26] Clark LN, Kartsaklis LA, Wolf Gilbert R, Dorado B, Ross BM (2009). Association of glucocerebrosidase mutations with dementia with lewy bodies.. Arch Neurol.

[27] Bras J, Singleton A, Cookson MR, Hardy J (2008). Emerging pathways in genetic Parkinson's disease: Potential role of ceramide metabolism in Lewy body disease.. Febs J.

[28] Mehrabian M, Castellani LW, Wen PZ, Wong J, Rithaporn T (2000). Genetic control of HDL levels and composition in an interspecific mouse cross (CAST/Ei×C57BL/6J).. J Lipid Res.

[29] Pattaro C, Marroni F, Riegler A, Mascalzoni D, Pichler I (2007). The genetic study of three population microisolates in South Tyrol (MICROS): study design and epidemiological perspectives.. BMC Med Genet.

[30] Vitart V, Biloglav Z, Hayward C, Janicijevic B, Smolej-Narancic N (2006). 3000 years of solitude: extreme differentiation in the island isolates of Dalmatia, Croatia.. Eur J Hum Genet.

[31] Rudan I, Marusic A, Jankovic S, Rotim K, Boban M (2009). “10001 Dalmatians:” Croatia launches its national biobank.. Croat Med J.

[32] Liebisch G, Drobnik W, Reil M, Trumbach B, Arnecke R (1999). Quantitative measurement of different ceramide species from crude cellular extracts by electrospray ionization tandem mass spectrometry (ESI-MS/MS).. J Lipid Res.

[33] Liebisch G, Lieser B, Rathenberg J, Drobnik W, Schmitz G (2004). High-throughput quantification of phosphatidylcholine and sphingomyelin by electrospray ionization tandem mass spectrometry coupled with isotope correction algorithm.. Biochim Biophys Acta.

[34] Aulchenko YS, Ripke S, Isaacs A, van Duijn CM (2007). GenABEL: an R library for genome-wide association analysis.. Bioinformatics.

[35] Devlin B, Roeder K (1999). Genomic control for association studies.. Biometrics.

[36] Amin N, van Duijn CM, Aulchenko YS (2007). A genomic background based method for association analysis in related individuals.. PLoS ONE.

[37] Chen WM, Abecasis GR (2007). Family-based association tests for genomewide association scans.. Am J Hum Genet.

[38] Dudbridge F, Gusnanto A (2008). Estimation of significance thresholds for genomewide association scans.. Genet Epidemiol.

